# Development of a high**-**throughput assay for rapid screening of butanologenic strains

**DOI:** 10.1038/s41598-017-18074-7

**Published:** 2018-02-21

**Authors:** Chidozie Victor Agu, Stella M. Lai, Victor Ujor, Pradip K. Biswas, Andy Jones, Venkat Gopalan, Thaddeus Chukwuemeka Ezeji

**Affiliations:** 1 Department of Animal Sciences, The Ohio State University, and Ohio State Agricultural Research and Development Center (OARDC), 305 Gerlaugh Hall, 1680 Madison Avenue, Wooster, OH 44691 USA; 2 Department of Chemistry and Biochemistry, and Center for RNA Biology, The Ohio State University, 484 West 12th Avenue, Columbus, OH 43210 USA; 3 Bioenergy and Biological Waste Management Program, Agricultural Technical Institute, The Ohio State University, 1328, Dover Road, Wooster, OH 44691 USA; 4 Present Address: Metahelix Life Sciences Limited, Bangalore, 560099 India

## Abstract

We report a *Thermotoga hypogea* (*Th*) alcohol dehydrogenase (ADH)-dependent spectrophotometric assay for quantifying the amount of butanol in growth media, an advance that will facilitate rapid high-throughput screening of hypo- and hyper-butanol-producing strains of solventogenic *Clostridium* species. While a colorimetric nitroblue tetrazolium chloride-based assay for quantitating butanol in acetone-butanol-ethanol (ABE) fermentation broth has been described previously, we determined that *Saccharomyces cerevisiae* (*Sc*) ADH used in this earlier study exhibits approximately 13-fold *lower* catalytic efficiency towards butanol than ethanol. Any *Sc* ADH-dependent assay for primary quantitation of butanol in an ethanol-butanol mixture is therefore subject to “ethanol interference”. To circumvent this limitation and better facilitate identification of hyper-butanol-producing *Clostridia*, we searched the literature for native ADHs that preferentially utilize butanol over ethanol and identified *Th* ADH as a candidate. Indeed, recombinant *Th* ADH exhibited a 6-fold *higher* catalytic efficiency with butanol than ethanol, as measured using the reduction of NADP^+^ to NADPH that accompanies alcohol oxidation. Moreover, the assay sensitivity was not affected by the presence of acetone, acetic acid or butyric acid (typical ABE fermentation products). We broadened the utility of our assay by adapting it to a high-throughput microtiter plate-based format, and piloted it successfully in an ongoing metabolic engineering initiative.

## Introduction

Growing concerns over the environmental consequences of fossil fuel use have reinvigorated interest in renewable energy sources like biofuels (e.g., bio-butanol). Solventogenic *Clostridium* species like *Clostridium beijerinckii* (*Cb*) are the only known natural and reliable producers of butanol. These microbes typically exhibit a biphasic metabolism wherein acetic and butyric acids produced during the acidogenic growth phase are re-assimilated to produce acetone, butanol, and ethanol (ABE) during the solventogenic phase. Current research on bio-butanol primarily focuses on maximizing production by butanologenic clostridia by enabling better utilization of commonly used biological feedstocks (e.g., lignocellulosic biomass). Such an approach relies heavily on the development of effective molecular tools for targeted metabolic engineering of desired phenotypes (e.g., high butanol production) in solventogenic *Clostridium* species^1^.

**Figure 1:**
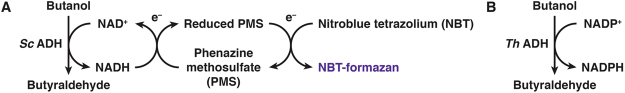
Schematic of *Sc* and *Th* ADH assays for n-butanol. (**A**) *Sc* ADH assay: the final spectrophotometric readout entails measuring purple NBT-formazan that absorbs at 580 nm and is generated upon reduction of NBT^1,2^. ADH-catalyzed oxidation of n-butanol provides the source of electrons (albeit via an intermediary, PMS) for this reduction. (**B**) *Th* ADH assay: the final spectrophotometric readout measures the absorbance of NADPH at 340 nm.

Even as various techniques for genetic manipulation of solventogenic *Clostridium* species are being fine-tuned to generate hyper-butanol-producing solventogenic *Clostridium* strains and to enable the characterization of genes crucial for butanol biosynthesis, it is essential to have an efficient and sensitive method for rapid screening of resultant mutant libraries to identify strains with desirable phenotypes. While gas chromatography is traditionally used to quantify concentrations of different components in complex mixtures like fermentation media^2,3^, it is unsuitable for high throughput analyses of large mutant libraries. Consequently, a colorimetric assay based on the NADH-coupled oxidation of nitroblue tetrazolium by commercially available *Saccharomyces cerevisiae* (*Sc*) alcohol dehydrogenase^4,5^ (ADH; Fig. 1A) was recently adapted by Scheel and Lütke-Eversloh^1^ to quantify the amount of butanol present in ABE fermentation media. In this study, we established that *Sc* ADH has a higher specificity for ethanol than butanol, and concluded that *Sc* ADH-based assays can only report on the *total* amount of alcohol in a sample and cannot serve as a stand-alone assay technique for butanol when present in an alcohol mixture. As a robust alternative for the rapid and efficient screening of engineered strains of butanologenic clostridia, we developed a robust and high-throughput microtiter plate-based spectrophotometric assay that utilizes recombinant *Thermotoga hypogea* (Th) ADH, an enzyme that prefers butanol over ethanol^6^ for rapid and efficient screening of engineered butanologenic clostridial strains.

## Materials and Methods

### Declaration of level of biocontainment

All bacteriological experimnents were conducted in compliance with the safety and biocontainment regulations associated with a biological safety levels one (BSL-1) laboratory.

### Strains and culture conditions

*Clostridium beijerinckii* NCIMB 8052 (*Cb*; ATCC 51743) and *Clostridium pasteurianum* (*Cp*; ATCC 6013) were obtained from the American Type Culture Collection (Manassas, VA). Laboratory stocks of these microorganisms were maintained as spore suspensions in sterile, double-distilled water at 4 °C. *Cb* spores were revived and propagated as previously described^7^. For inoculation, *Cp* spores (200 µL) were heat-shocked at 75 °C for 10 min, cooled on ice for 2 min, and then inoculated into 10-mL anoxic tryptone–glucose–yeast extract (TGY) media. The culture was then incubated in an anaerobic chamber (Coy Laboratory Products Inc., Ann Arbor, MI) with a modified atmosphere of 82% N_2_, 15% CO_2_, and 3% H_2_ at 35 °C until it reached an OD_600_ ~ 0.9–1.1^7^. Actively growing *Cp* (10%, v/v) was subsequently transferred to fresh TGY media (90 mL) and incubated for 3–4 h (until the culture reached an OD_600_ ~1.1) to increase the pre-culture volume.

### Determination of kinetic parameters for *Sc* alcohol dehydrogenase (ADH) and effect of other fermentation products on n-butanol quantitation

The kinetic parameters for *Sc* ADH were determined by monitoring the substrate-dependent change in the absorbance of NBT-formazan at 580 nm. During the oxidation of alcohols to their respective aldehydes by *Sc* ADH, NADH is formed, which reduces NBT in the presence of phenazine methosulfate (PMS) to form NBT-formazan (Fig. 1A). *Sc* ADH-based assays (100 µl each) were performed in 1X Sc buffer (100 mM Tris-HCl, pH 8.6; 330 µM NAD^+^; 8 µM PMS; 330 µM NBT; 0.1% gelatin, which stabilizes the colloidal NBT-formazan product). For each reaction, 37.3 nM *Sc* ADH (0.1 µl of 28 µM stock; A7011; Sigma Aldrich, St. Louis, MO) was incubated for 5 min at ~22 °C in 75 µl 1.33X Sc buffer. Alcohol oxidation was initiated by transferring the pre-incubated enzyme to the well of a microtiter plate (96-well Immulon® HBX MicrotiterTM plate; Thermo Scientific, Waltham, MA), which contained 25 µl appropriate substrate mixture. Absorbance at 580 nm was then measured at 5-min intervals for 30 min using a FlexStation 3 Multi-Mode Microplate Reader (Molecular Devices, Sunnyvale, CA).To determine the kinetic parameters for *Sc* ADH with either ethanol or n-butanol as the sole substrate, various concentrations of ethanol [0 to 10 g/L (or 217 mM)] and n-butanol [0 to 50 g/L (or 675 mM)] were tested. Absorbance values were plotted as a function of time, and the slope of the best fit line for each plot was then divided by the molar extinction coefficient of NBT-formazan at 580 nm (12,300 M^−1^ cm^−1^)^4,8^ to determine initial velocity (expressed as mmol NBT-formazan/min).

**Figure 2:**
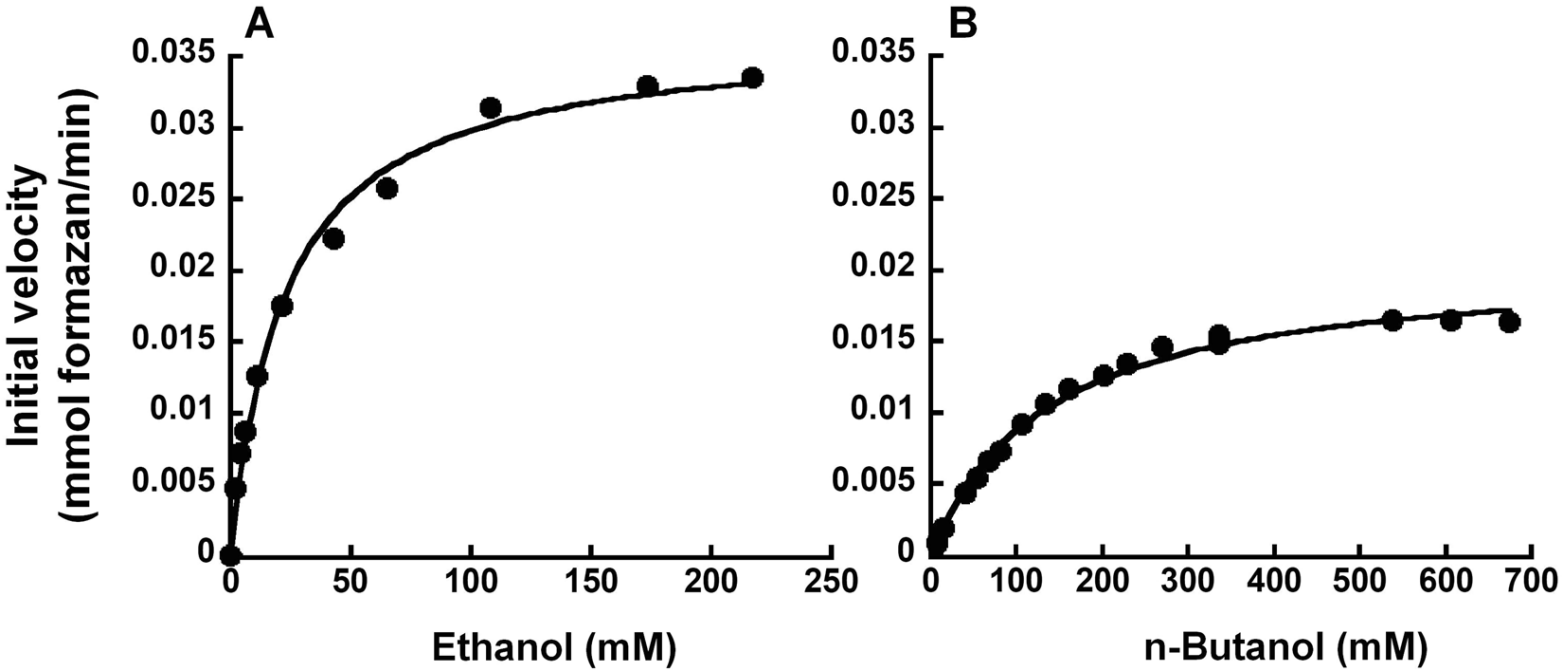
Michaelis-Menten analyses of *S. cerevisiae* ADH-catalyzed oxidation of ethanol (**A**) and n-butanol (**B**). While representative plots are depicted, the k_cat_ and K_m_ values reported in the text represent the mean and standard deviation calculated from four independent experiments. The curve-fit errors in individual trials did not exceed 14.9% (K_m_, ethanol), 4.4% (V_max_, ethanol), 8.7% (K_m_, butanol) and 3.6% (V_max_, butanol). R^2^ values of individual trials exceeded 0.99.

To evaluate the effect of ethanol on the activity of *Sc* ADH with n-butanol as the primary substrate, assays were conducted using substrate mixtures containing varying concentrations of ethanol (0.3, 0.5, 0.8, 1, or 2 g/L)–chosen based on the linear portion of the appropriate Michaelis-Menten curve (Fig. 2A)–and a fixed concentration of n-butanol (6 g/L or 81 mM)– roughly the K_m_ of *Sc* ADH for n-butanol (Fig. 2B). To ascertain the effect of other major ABE fermentation products on the activity of *Sc* ADH with n-butanol as the primary substrate, the enzyme was assayed using substrate mixtures containing (A) n-butanol and acetone or (B) n-butanol and either acetic or butyric acid in ratios typically found in ABE fermentation media; thus, the concentrations tested in each substrate mixture were (A) 0.6 and 0.3 g/L; 1.2 and 0.6 g/L; 4 and 2 g/L; or 6 and 3 g/L; and (B) 1.2 and 0.15 g/L; 3 and 0.375 g/L; 4 and 0.5 g/L; or 6 and 0.75 g/L. Mean and standard deviation values were calculated from three independent replicates of each assay.

### Cloning, overexpression, and purification of *Th* ADH

The *Th* ADH open reading frame (ORF) was identified by using tBLASTn (National Center for Biotechnology Information) to search the *Th* genome for the N-terminal sequence of *Th* ADH (**MENFVFHNPTKLIFG**), which was experimentally determined using mass spectrometry by Ying *et al*.^6^. The *Th* ADH gene was amplified by PCR using as template *Th* genomic DNA (gDNA), which was kindly provided by Dr. Kesen Ma, University of Waterloo, Waterloo, Ontario, Canada. After various trials, successful amplification was achieved using CloneAmp HiFi PCR Premix (Clontech) with *Th*ADH-2F (5′-CATCAGCGATCCA TGGAAAACTTCGTTTTTCAC-3′) and *Th*ADH-R (5′-CTTGGGAAGCTAT GAGCAGGATCTG-3′) as primers. The underlined sequences in the primers facilitated cloning of the PCR amplicon into the T7 RNA polymerase-driven expression vector pET-33b at the *Nco*I and filled-in *Xho*I sites to create pET-33b–*Th* ADH, whose sequence was confirmed using automated DNA sequencing at the OSU Comprehensive Cancer Center Genomics Facility. In addition to T7 promoter and terminator primers, *Th*ADH_internal-F (5′-GCAGGTAGGAAA GGTGGGGATTGG-3′) was used to ensure complete sequencing coverage of the *Th* ADH insert. Note that pET-33b–*Th* ADH encodes an N-terminal His_6_-tagged *Th* ADH.

Overexpression of *Th* ADH in *Escherichia coli* (Ec) BL21 (DE3) Rosetta cells was conducted anaerobically while enzyme purifications were either performed aerobically to yield *Th* ADH_ae or anaerobically to yield *Th* ADH_an. For overexpression of *Th* ADH, Ec BL21 (DE3) Rosetta cells were transformed with pET-33b–*Th* ADH, and 5 mL LB media containing 35 µg/mL kanamycin and 35 µg/mL chloramphenicol was inoculated with a single colony and subsequently grown aerobically for ~16 h at 37 °C with shaking (225 RPM). This overnight seed culture was used to inoculate 2 L of deoxygenated LB media supplemented with 2 mM cysteine, 0.2% (w/v) glucose, and antibiotics as listed above. The large-scale culture was then grown under strict anaerobic conditions in a chamber with a modified atmosphere of 95% N_2_ and 5% H_2_ at 37 °C with shaking (100 RPM) for ~12 h until it reached an OD_600_ ~ 0.6. Protein overexpression was then induced with 1 mM isopropyl-β-D-thiogalactoside, and the culture was anaerobically grown for an additional 12 h at 37 °C with shaking (100 RPM). Cells were harvested by centrifugation, and purification was initiated immediately using a freshly harvested 1-L cell pellet.

*Th* ADH_ae and *Th* ADH_an were largely purified in the same manner. However, to prevent oxidation of *Th* ADH_an, all purification steps were conducted under strict anaerobic conditions in the same chamber used for overexpression; stock solutions and purification buffers were also placed in the chamber for four days to allow for complete deoxygenation prior to use. To begin purification, the 1-L cell pellet was resuspended in 20 mL of Buffer L [50 mM Tris-HCl, pH 7.5; 300 mM NaCl; 50 mM imidazole; 5 mM β-mercaptoethanol (β-ME); one cOmpleteTM Mini, EDTA-free, protease inhibitor cocktail tablet (Roche, Basel, Switzerland); 0.1 mM phenylmethylsulfonyl fluoride (PMSF); 40 U DNase I (Roche); 0.5 mg/mL lysozyme (Gold Biotechnology, St. Louis, MO)] and then sonicated. Following centrifugation (24,000 g; 20 min; 4 °C), the supernatant was passed through a 0.22-µm syringe filter (Millipore, Burlington, MA), applied to a pre-equilibrated 1-mL HisTrap HP column (GE Healthcare, Chicago, IL), and eluted with a step-wise 50–500 mM imidazole gradient in Buffer B (50 mM Tris-HCl, pH 7.5; 300 mM NaCl; 500 mM imidazole; 5 mM β-ME; 0.1 mM PMSF) using an ÄKTA FPLC purifier (GE Healthcare). Fractions containing *Th* ADH, which eluted between ~190 to 255 mM imidazole, were identified using SDS-PAGE, pooled, supplemented with 10% (v/v) glycerol, aliquoted, and stored at −80 °C. Protein concentration was quantified using absorbance at 280 nm and *Th* ADH’s molar extinction coefficient of 54,850 M^−1^cm^−1^.

### *Thermotoga hypogea* ADH_ae-dependent n-butanol time-course assays

The kinetic parameters for recombinant *Th* ADH_ae were determined as previously described^6^ by monitoring the substrate-dependent change in the absorbance of NADPH at 340 nm; NADP^+^ was reduced during the oxidation of alcohols to their appropriate aldehydes by *Th* ADH (Fig. 1B). Given the oxygen-sensitive nature of the iron-dependent enzyme, *Th* ADH_ae-based assays (125 µl each) were conducted anaerobically at 80 °C in 1X Th buffer [200 mM N-cyclohexyl-3-aminopropanesulfonic acid (CAPS), pH 11.6; 1 mM DTT; 0.1 mM FeCl_2_]. For each reaction, 344 nM *Th* ADH_ae (3 µl of 10 µM stock) was incubated for 10 min at 30 °C with 1.56X Th buffer in a volume of 80 µl before 45 µl appropriate substrate mixture supplemented with 1.33 mM NADP^+^ was added. Subsequently, 25 µl of this assay mixture was immediately dispensed into each of five 1.5-mL tubes and incubated at 80 °C for 0, 20, 40, 60, or 80 s before the reaction was terminated with an equal volume of quench solution [1 M sodium acetate, pH 4.5; 1 mM EDTA]. Each reaction was then transferred to a 96-well microtiter plate, and absorbances at 340 nm were measured using an Epoch Microplate Spectrophotometer (BioTek Instruments, Inc., Winooski, VT). The stepwise protocol for this assay is provided in Table S1.

**Figure 3:**
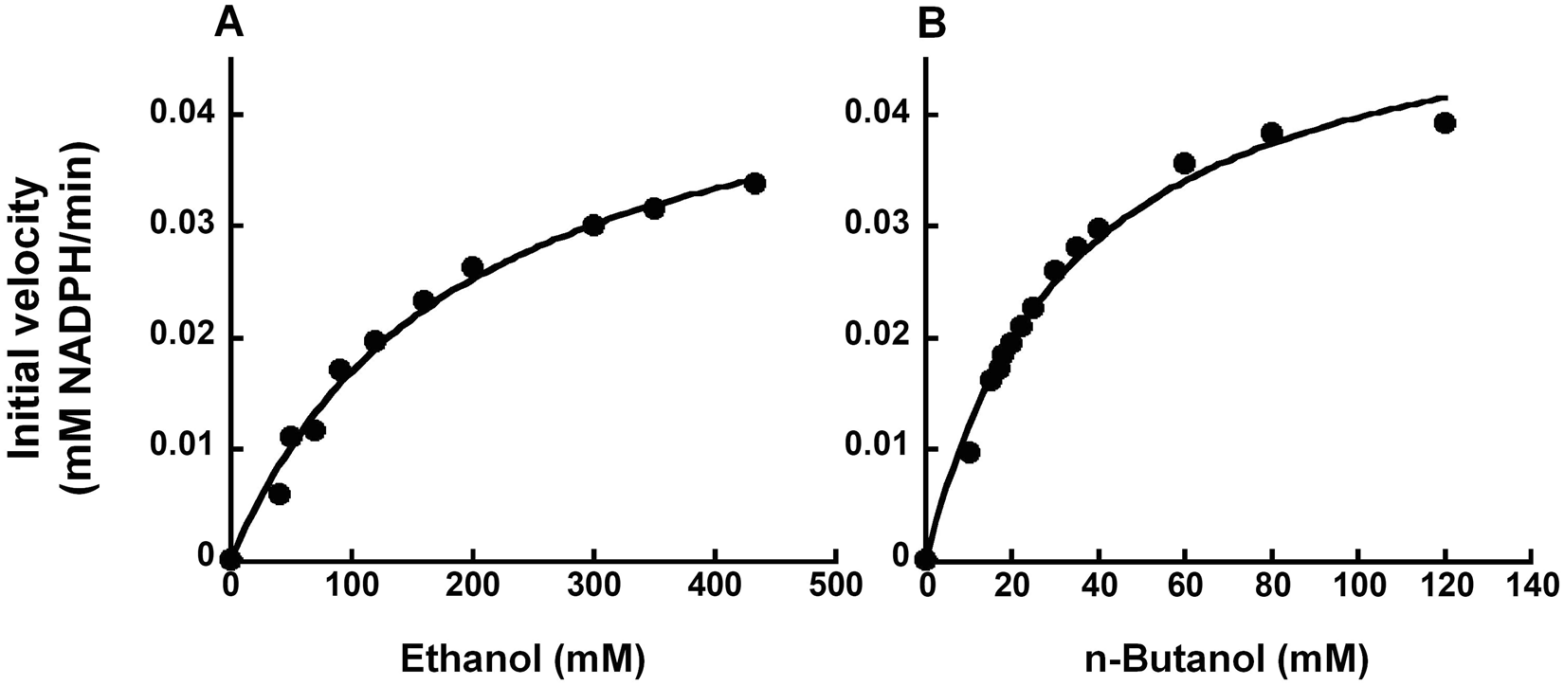
Michaelis-Menten analyses of *Th* ADH_ae-catalyzed oxidation of ethanol (**A**) and n-butanol (**B**). While representative plots are depicted, the k_cat_ and K_m_ values reported in the text represent the mean and standard error calculated from two independent experiments. The curve-fit errors in individual trials did not exceed 16.5% (K_m_, ethanol), 8% (V_max_, ethanol), 15% (K_m_, butanol) and 7% (V_max_, butanol). R^2^ values of individual trials exceeded 0.98.

The kinetic parameters for *Th* ADH_ae were determined using a variety of different substrate mixtures, which were prepared largely as described for the *Sc* ADH-based assays. In reactions where ethanol or n-butanol was the only substrate present, the concentrations that were tested ranged from 0 to 20 g/L (or 434 mM) or 0 to 8.9 g/L (or 120 mM), respectively. Initial velocities (expressed as mM NADPH/min) were calculated using the molar extinction coefficient of NADPH at 340 nm (6,220 M^−1^cm^−1^). To evaluate the extent of ethanol interference, substrate mixtures were prepared with varying concentrations of ethanol (0.4, 0.5, 0.8, 1, 2, or 5 g/L)–selected from the linear portion of the appropriate Michaelis-Menten curve (Fig. 3A)–and a fixed concentration of n-butanol (2.6 g/L)–roughly the K_m_ of *Th* ADH_ae for n-butanol (Fig. 3B). To determine whether the presence of other major ABE fermentation products affected *Th* ADH activity, substrate mixtures contained (A) n-butanol and acetone or (B) n-butanol and either acetic or butyric acid in ratios commonly found in ABE fermentation media (~2.6:1 and ~7.4:1, respectively); the concentrations tested in each substrate mixture were (A) 0.74 and 0.3 g/L; 1.1 and 0.4 g/L; 1.5 and 0.6 g/L; or 3 and 1.2 g/L; and (B) 0.74 and 0.1 g/L; 1.1 and 0.15 g/L; 1.5 and 0.2 g/L; or 3 and 0.4 g/L. Mean and standard error of the mean were determined from two independent assays.

### Generation of recombinant glycerol dehydrogenase (*dha*D1 and *gld*A1) and dihydroxyacetone kinase (*dhaK*) constructs

**Figure 4:**
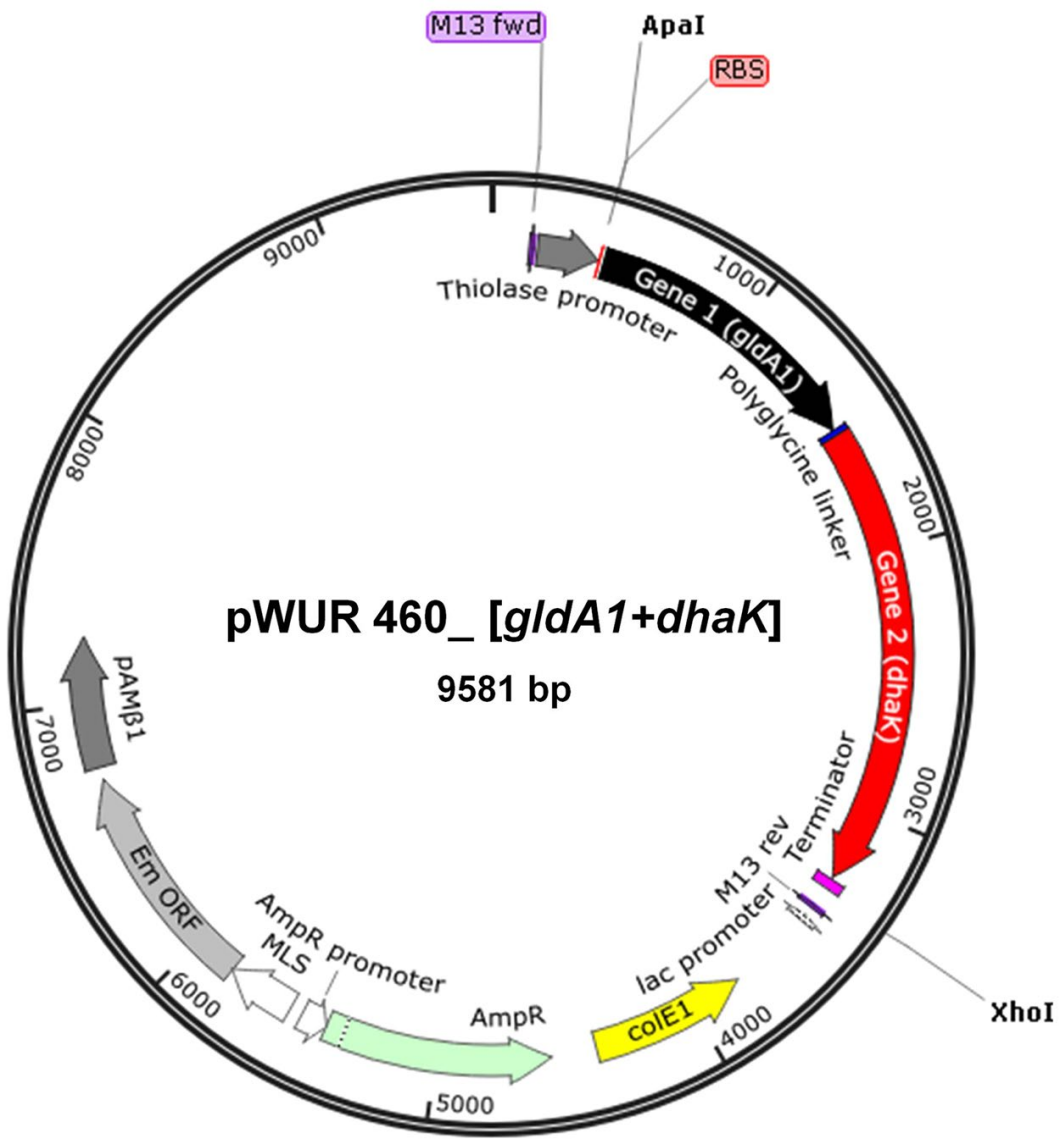
Schematic of recombinant plasmids (pWUR460_*dhaD1* + *gldA1* or pWUR460_*gldA1* + *dhaK*) used to transform electrocompetent *C. beijerinckii*. Gene expression was driven by the constitutive *C. acetobutylicum* thiolase promoter (P_*Thl*_)^10^. See text for details of fused gene constructs.

Gene sequences for *Cp* *dhaD1, gldA1*, and *dhaK* were obtained from the EMBL-European Bioinformatics Institute. Each gene was subsequently amplified from *Cp* gDNA using nested PCR while splicing by overlap extension (SOE) PCR was used to generate the final fused gene constructs. All PCR reactions were performed using PrimeSTAR GXL DNA polymerase (Clontech, Mountain View, CA) and an iCycler^TM^ Thermal Cycler (Bio-Rad, Hercules, CA). Each 50-µL reaction contained 1X PrimeSTAR GXL buffer, 0.25 mM dNTPs, 0.5 µM primers, ~5 ng/µL DNA template, and 1.25 U PrimeSTAR GXL DNA polymerase. Primer sequences and annealing temperatures (ATs; with AT1 for the portion of the primer complementary to the DNA template and AT2 for the entire primer sequence) are listed in Table S2. Primers were designed to introduce a Gly5 peptide linker and a ribosome-binding site and to facilitate cloning of the final fused gene construct into the *Clostridium*-Ec shuttle plasmid pWUR460 at either the 5′ ApaI or NcoI site and the 3′ EcoRI or XhoI site (Fig. 4).

The cycling conditions for the nested PCR reactions that were used to amplify each gene from *Cp* gDNA were: 98 °C for 2 min (initial denaturation); five cycles of 98 °C for 20 s, AT1 for 20 s, 72 °C for 30 s and 30 cycles of 98 °C for 20 s, AT2 for 20 s, 72 °C for 30 s (denaturation, annealing, and extension; 72 °C for 5 min (final extension); and 4 °C for 10 min.

A modified two-step SOE-PCR technique^9^ was then used to build the final fused gene constructs. Gene 1 and gene 2 PCR amplicons (e.g., *dhaD1* and *gldA1*) from the nested PCR reactions were used as the templates (forward- and reverse- templating fragments, respectively) for Step 1 SOE-PCR, which generates the fused gene inserts (i.e., [*dhaD1* + *gldA1*] or [*gldA1* + *dhaK*]). Cycling conditions for Step 1 SOE-PCR were: 98 °C for 2 min; 15 cycles of 98 °C for 1 min, AT of overlap region (nucleotide sequence in the region where the two genes will be linked) for 2 min, 72 °C for 3 min; 72 °C for 10 min; and 4 °C for 10 min. The forward primer for gene 1 (e.g., dhaD1-F) and the reverse primer for gene 2 (e.g., gldA1-R) were then added to the Step 1 SOE-PCR reaction, and the following cycling conditions were used for Step 2 SOE-PCR: 98 °C for 2 min; 30 cycles of 98 °C for 1 min, AT of the forward primer for gene 1 (i.e., dhaD1-F or gldA1-F) for 2 min, 72 °C for 3 min; 72 °C for 5 min; and 4 °C for 10 min. Each insert (i.e., *dhaD1* + *gldA1* or *gldA1* + *dhaK*) was then spliced into the *Clostridium- E. coli* shuttle plasmid pWUR460 to permit transcription by a constitutive thiolase promoter from *C. acetobutylicum*^10^. A general schematic of the final recombinant plasmids is depicted in Fig. 4.

### Transformation of electrocompetent *Clostridium beijerinckii* (*CB*) cells and butanol fermentation

Electrocompetent *Cb* cells were transformed to generate two recombinant strains: *Cb*-pWUR460_*dhaD1* + *gldA1* and *Cb*-pWUR460_*gldA1* + *dhaK*. To prepare electro-competent *Cb* cells, 200 µL of *Cb* spores were heat-shocked at 75 °C for 10 min, cooled on ice for 2 min, and then inoculated into 10 mL anoxic tryptone–glucose–yeast extract (TGY) medium^11^. The culture was grown at 35 °C in an anaerobic chamber with a modified atmosphere of 82% N_2_, 15% CO_2_, and 3% H_2_ as previously described^11^ until it reached an OD_600_ ~ 0.9–1.1 (usually ~12 h from time of inoculation). The culture was then plated onto TGY agar [0.5% (w/v)] and incubated until single colonies appeared. A single colony was subsequently inoculated into 10 mL TGY liquid media and incubated for 10 h. Actively growing *Cb* cells [10% (v/v)] were then transferred into 90 mL of fresh TGY media and incubated until an OD_600_ ~0.6–0.8. Cells were harvested by centrifugation at 4,000 g and 4 °C for 6 min. Cell pellets were washed once with 50 mL of electroporation buffer [5 mM KH_2_PO_4_; 270 mM sucrose; 1 mM MgCl_2_, 10% (w/v) PEG-8000] and then resuspended in 2 mL of electroporation buffer before incubation on ice for 5 min.

To transform *Cb*, 10 µg of plasmid DNA was gently mixed with 400 µL freshly prepared *Cb* electrocompetent cells kept in a pre-chilled 0.2-cm electroporation cuvette. Electroporation was conducted inside the anaerobic chamber using a Bio-Rad Gene Pulser Xcell Electroporator with the following setting: 2.5 kV, 25 µF capacitance, and infinite resistance^12^; pulse delivery varied between 2.9 and 4.2 msec. After electroporation, cells were diluted in 4 mL of TGY media and incubated anaerobically at 35 °C for 6 h to allow cell recovery and expression of the antibiotic resistance gene. Recovered cells were pelleted at 3,000 g for 5 min, mixed with semi-solid TGY agar containing 25 µg/mL erythromycin, and then incubated in an anaerobic chamber for 48–72 h. Colonies were picked, transferred into fresh TGY agar containing 25 µg/mL erythromycin, and grown for another 12–24 h. A total of 24 single colonies were randomly picked from the two *Cb* transformants (15 colonies [**A–O**] from pWUR460_[*dhaD1* + *gldA1*] and nine colonies [**P–X**] from pWUR460_[*gldA1* + *dhaK*]). Single colonies were inoculated into different wells of a microtiter plate that contained 200 µL of 30 g/L tryptone, 36 g/L glucose, 36.1 g/L glycerol, 10 g/L yeast extract, and 25 µg/mL erythromycin and then incubated at 35 °C in the anaerobic chamber. After 60 h of growth, the microtiter plate was centrifuged at 2,500 g for 2 min. The supernatants were diluted five-fold and then screened for butanol using the high-throughput platform described below.

### Development of *a Th* ADH-based high throughput platform to screen *Cb* cultures for n-butanol: microtiter plate-based end-point assays

To develop a multiplex platform for the rapid screening of large *Clostridium* libraries for n-butanol, end-point assays were developed. To ensure uniform heat distribution throughout the reaction, multi-well PCR plates and a Bio-Rad iCycler^TM^ Thermal Cycler were used to incubate the samples for 80 s. To minimize pipetting errors, two master mixes and a multichannel pipette were used as described below.

Master Mix 1 contained 25 µL of 500 mM CAPS, pH 11.6; 6.25 µL of 10 mM DTT; and 6.25 µL of 1 mM FeCl_2._ Master Mix 2 contained 15 µL of 2 mM NADP^+^ and 1.5 µL 10 µM *Th* ADH. To screen for n-butanol in the 24 recombinant *Cb* cultures, 7.5 µL of each diluted supernatant described above were aliquoted into different wells of a fresh 96-well PCR plate + 1 µL distilled water inside an anaerobic chamber. Using a multichannel pipette, 37.5 µL of Master Mix 1 was added to each well before 16.5 µL of Master Mix 2 was added for a total assay volume of 62.5 µL (Table S1). To generate n-butanol standard curve, 7.5 µL samples with known concentrations of n-butanol (10 to 25 mM, a range chosen based on the linear part of the Michaelis-Menten curve) were tested alongside unknown *Cb* samples in the 96-well PCR plate. The PCR plate was then sealed and incubated in a thermocycler set at 80 °C. After 80 s, samples were transferred to a flat-base 96-well Immulon 4 HBX Microtiter™ plate for absorbance measurements at 340 nm. The concentration of butanol in each of the samples was calculated based on the standard curve and compared with results obtained from gas chromatography analysis.

We compared two types of high-throughput assays: assembling the reaction mixture inside either an anaerobic chamber (anaerobic) or on a standard laboratory workbench (aerobic); in both instances, the 96-well PCR plate was sealed prior to the 80 °C incubation for 80 s. In addition, we investigated potential variations from use of *Th* ADH purified under either aerobic (*Th* ADH_ae) or anaerobic (*Th* ADH_an) conditions in these two assays (Table 3). The following n-butanol concentrations were used as part of this comparison: 0, 17, 40, 80, 100, 125, 167, 180, 200, 250, 330, 400, and 500 mM.

### Gas chromatography analysis for n-butanol

Gas chromatography analysis of n-butanol in *Cb* culture supernatants was performed using an Agilent 7890 A gas chromatograph (Agilent Technologies, Inc., Wilmington, DE, USA) equipped with a flame ionization detector (FID) and a J&W capillary column [19091 N-213; 30 m (length) × 320 μm (internal diameter) × 0.50 μm (HP-INNOWax film)]^11^.

### Statistical analysis

The general linear model of Minitab version 17 (Minitab, Inc., State College, PA) was used for all statistical comparisons of kinetic parameters: initial velocity, V_max_, K_m_, k_cat_, and k_cat_/K_m_. Turnover number (k_cat_) is defined as the number of substrate molecules converted to product per catalytic site per unit time (V_max_/[E]) while the specificity constant (k_cat_/K_m_) indicates the catalytic efficiency of the enzyme. Fisher’s least significant difference (LSD) at a 95% confidence interval was applied for pairwise comparisons of mean values.

## Results and Discussion

### *Sc* ADH exhibits higher activity towards ethanol than butanol

A colorimetric assay exploiting the NADH-coupled oxidation of nitroblue tetrazolium by *Sc* ADH to quantify total alcohol concentrations has been reported^4^. This assay was subsequently adapted by Scheel and Lütke-Eversloh^1^ to measure the total alcohol present in ABE fermentation media in a high-throughput format. To ascertain the preference of *Sc* ADH for either ethanol or butanol, we used a formazan-based readout to experimentally determine the kinetic parameters of the enzyme for both substrates. Figure 2 shows a representative Michaelis-Menten plot for *Sc* ADH with either ethanol (Fig. 2A) or butanol (Fig. 2B) as the substrate. The V_max_ and K_m_ for ethanol were determined from non-linear curve fitting to be 0.034 ± 0.004 mM min^−1^ and 22.7 ± 2.9 mM, respectively, while the V_max_ and K_m_ for butanol were determined to be 0.018 ± 0.002 mM min^−1^ and 158 ± 34 mM, respectively. The K_m_ of *Sc* ADH for ethanol is similar to the 21 mM reported by the commercial supplier (Sigma). Turnover numbers (k_cat_) for *Sc* ADH on ethanol and butanol were 1230 ± 142 min^−1^ and 651 ± 63 min^−1^, respectively, while catalytic efficiencies (k_cat_/K_m_) were 54 ± 3 min^−1^mM^−1^ and 4.3 ± 1.2 min^−1^mM^−1^, respectively. The higher turnover number (~2-fold) and catalytic efficiency (~13-fold) observed with ethanol compared to butanol reveals a clear preference of *Sc* ADH for the former, an attribute that has long been recognized^13–15^ but validated here largely to facilitate comparisons with other ADH variants under similar assay conditions.

### Ethanol interferes with butanol measurements during *Sc* ADH assays

While the *Sc* ADH-based assay can be used to accurately quantify butanol when it is not present in an alcohol mixture, we hypothesized on the basis of our kinetic data that the presence of ethanol could interfere with butanol measurements given the enzyme’s higher activity toward ethanol than butanol. To test this idea, initial velocities of *Sc* ADH with different ratios of ethanol + butanol mixtures were compared to the initial velocity of the enzyme with pure butanol. Substrate mixtures contained a fixed concentration of butanol (6 g/L) and varying concentrations of ethanol (0.3, 0.5, 0.8, 1, and 2 g/L). By design, these concentrations spanned the range of butanol and ethanol concentrations produced by solventogenic *Clostridium* species during normal ABE fermentation.

**Figure 5:**
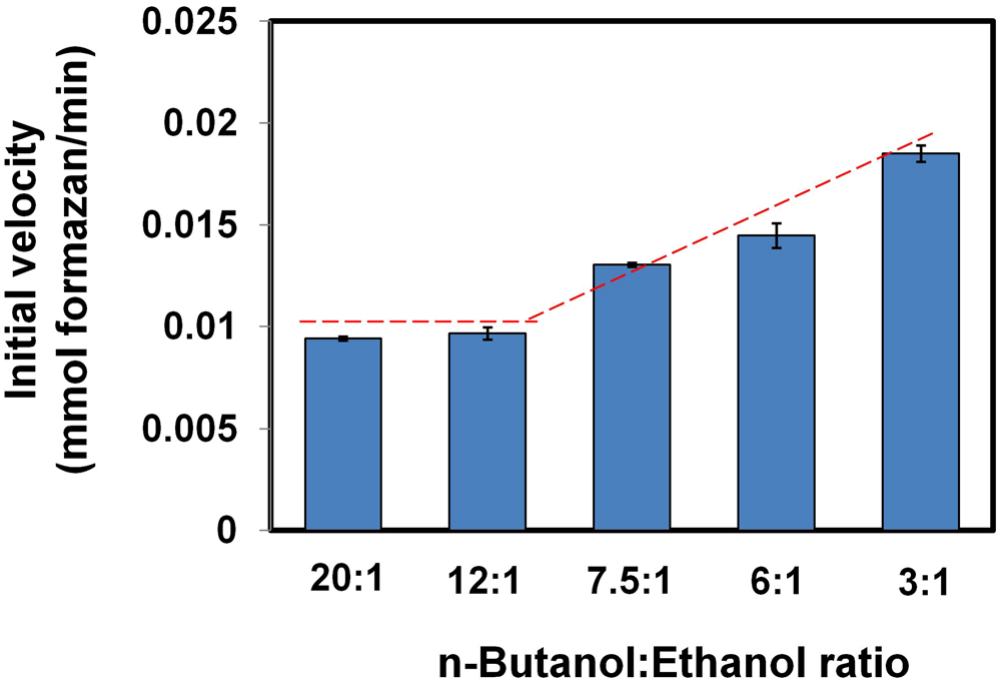
Ethanol interference of *S. cerevisiae* ADH activity towards n-butanol. Initial velocities were determined in mixtures containing a fixed amount of n-butanol (6 g/L) and varying concentrations of ethanol (0.3, 0.5, 0.8, 1, and 2 g/L). The dashed line indicates change in initial velocity as the concentration of ethanol increases. The initial velocity of ethanol was not altered when the butanol to ethanol ratio is at least 12:1.

Figure 5 shows that the initial velocity of *Sc* ADH remained constant when the relative butanol concentration in the mixture was significantly higher than the relative concentration of ethanol. When butanol was present in either a 20:1 or a 12:1 ratio to ethanol, the initial velocities were similar, 0.0094 and 0.0097 mM formazan per min (*p* > 0.05), respectively, which suggests that the activity of *Sc* ADH with butanol at these ratios is not affected by the presence of ethanol and that absorbance measurements were solely due to butanol. However, at 8:1, 6:1, and 3:1 butanol:ethanol ratios, we observed a linear increase in the initial velocities −0.013, 0.015, and 0.019 mM formazan per min, respectively. Given the higher affinity of *Sc* ADH for ethanol compared to butanol, this result is not unexpected. Clearly, *Sc* ADH activity in butanol:ethanol mixtures is a mosaic, which cannot be unambiguously parsed to determine the concentration of the constituent alcohols.

**Figure 6:**
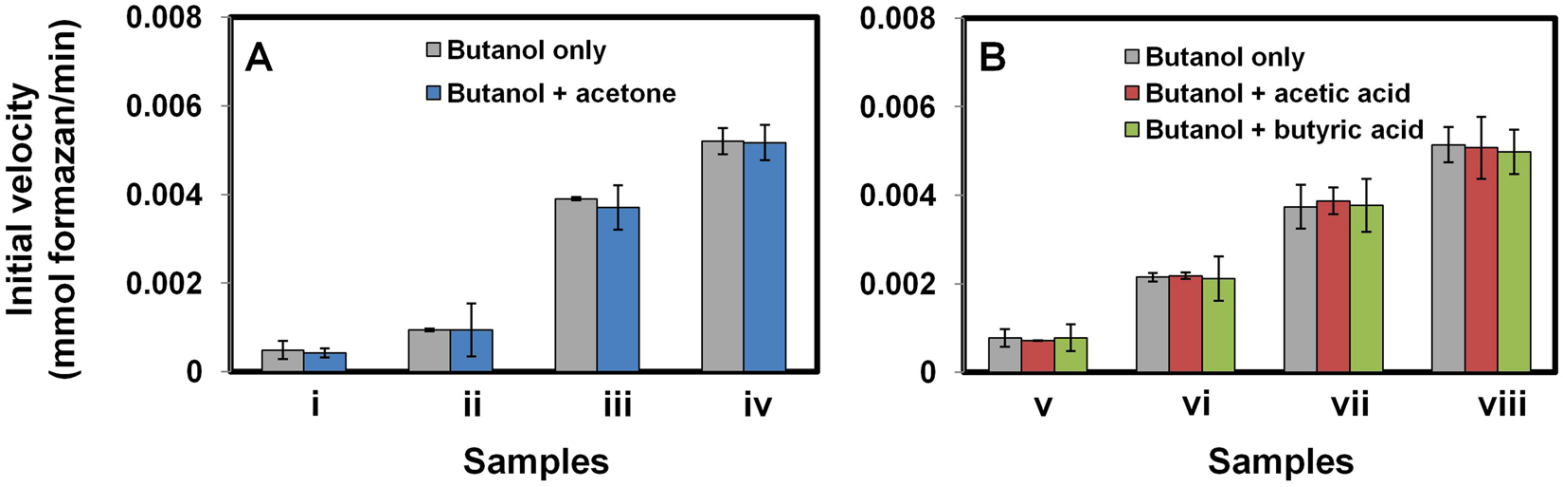
Effect of fermentation products on n-butanol activity of *S. cerevisiae* ADH. (**a**) acetone, (**b**) acetic and butyric acids. n-Butanol + acetone substrate mixtures consist of: (i) 0.6 and 0.3 g/L; (ii) 1.2 and 0.6 g/L; (iii) 4 and 2 g/L; (iv) 6 and 3 g/L, respectively. n-Butanol + acetic or butyric acids substrate mixtures consist of: (v) 1.2 and 0.15 g/L; (vi) 3 and 0.375 g/L; (vii) 4 and 0.5 g/L; (viii) 6 and 0.75 g/L.

Figure 6 shows the effect of acetone, acetic acid, or butyric acid on the activity of *Sc* ADH when butanol is present as the primary substrate. Initial velocity for *Sc* ADH using butanol in the absence of any additive was compared to that observed in the presence of acetone (in a 2:1 ratio; Fig. 6A) or either acetic acid or butyric acid (in a 8:1 ratio; Fig. 6B). These comparisons show conclusively that *Sc* ADH activity with butanol is not affected by the presence of acetone, acetic acid, or butyric acid.

### Design of a *Th* ADH-based assay

A review of the current literature (summarized in Table 1) reveals that assays developed with *Sc* ADH and other enzymes fail to preferentially quantify butanol in alcohol mixtures, although certain *Sc* ADH mutants with altered substrate specificity have not been fully explored^14,15^. Therefore, to develop a new, high-throughput assay for specific and accurate quantitation of butanol in fermentation media, we searched the literature for native ADHs with higher reported activity toward butanol than ethanol. This search led us to identify *Thauera butanivorans* (*Tb*) butanol dehydrogenase (BDH) and *Thermotoga hypogea* (*Th*) ADH as putative candidates: *Thauera butanivorans* (*Tb*) butanol dehydrogenase (BOH)^16,17^ and *Thermotoga hypogea* (*Th*) ADH^3^ exhibit 2.5- and 4-fold higher activity with butanol than ethanol, respectively. Because numerous challenges impeded attempts to develop an assay using *Tb* BOH, we focused our subsequent efforts on *Th* ADH.

**Table 1:** Overview of enzymatic assays used to screen for alcohol in biological samples.

	**References**	**Description of assay**
1.	Commercial alcohol assay kits (e.g., Emit II Plus ethyl alcohol assay kit from Beckman Coulter)	These assays are based on the conversion of NAD^+^ to NADH, which accompanies the oxidation of ethanol to acetaldehyde by *S. cerevisiae* ADH. The increase in absorbance at 340 nm is proportional to the concentration of alcohol in the sample. The assay measures total alcohol in samples.
2.	Scheel and Lütke-Eversloh^1^	This assay uses *S. cerevisiae* ADH and NBT-based quantitation of alcohol. Assay was successfully adapted to measure total alcohol in ABE fermentation, but cannot preferentially detect butanol (Fig. 1).
3.	Azevedo *et al*.^19^	Most alcohol oxidase (AOX)-based ethanol sensors monitor O_2_ consumption or H_2_O_2_ formation using amperometric electrodes (− 600 mV for O_2_ or + 600 mV for H_2_O_2_).
4.	Mangos and Haas^20^	A colorimetric assay employed to detect methanol using AOX, peroxidase, and 2,2′-azinobis(3-ethylbenzthiazoline-6-sulfonic acid) (ABTS).
5.	Verduyn *et al*.^21^	An assay based on a modified AOX from *Hansenula polymorpha*, which lack 90% its catalase activity, an essential attribute for colorimetric alcohol assays that are centered on peroxidase-mediated oxidation of dyes. Assay is particularly suitable for determination of ethanol in fermentation broths.
6.	This study	Employs a recombinant ADH from *T. hypogea*. This assay is capable of preferential quantitation of butanol in mixed alcohol substrate, including ABE fermentation broths.

**Figure 7:**
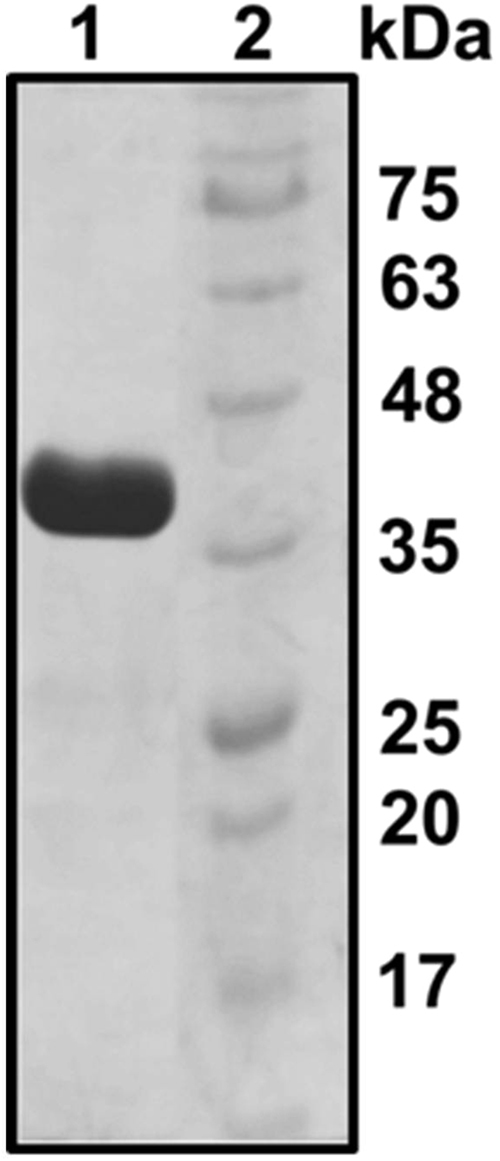
Purity of recombinant *Th* ADH_ae. SDS-PAGE [12% (w/v) polyacrylamide] analysis of the final ADH preparation obtained after overexpression in *E. coli* and affinity chromatography. Lane 1, 10 µg of purified ADH; lane 2, size markers.

Following cloning and overexpression in Ec BL21 (DE3) Rosetta cells, *Th* ADH was purified using immobilized metal-affinity chromatography either under aerobic or anaerobic conditions. SDS-PAGE [12% (w/v) polyacrylamide] of the final purified sample (from the anaerobic run) revealed the successful purification of a ~42 kDa protein, which corresponds to the expected mass of *Th* ADH (Fig. 7). Because the native *Th* ADH is highly sensitive to oxygen^6^, activity assays were conducted inside an anaerobic chamber. The initial kinetic studies were performed using ADH_ae (purified under aerobic conditions); we discovered later that the catalytic efficiency is similar for *Th* ADH_ae and *Th* ADH_an (i.e., enzyme purified under anaerobic conditions) (see below; Table 3). To evaluate the substrate preference of recombinant *Th* ADH_ae, time-course assays were used to determine its kinetic parameters for ethanol (Fig. 3A) and butanol (Fig. 3B). Initial velocities at different substrate concentrations were used to obtain Michaelis-Menten plots with butanol or ethanol as substrates. V_max_ for ethanol and butanol were 0.049 ± 0.0002 and 0.052 ± 0.001 mM NADPH per min, respectively, while the K_m_ for butanol (34.1 ± 0.6 mM) was 6-fold lower than that for ethanol (198 ± 8.9 mM); the K_m_ trend is a near reversal of the pattern observed with *Sc* ADH. The k_cat_ values for ethanol and butanol were 223.6 ± 0.9 min^−1^ and 236.6 ± 5.2 min^−1^, respectively. The specificity constants (k_cat_/K_m_) were 1.13 ± 0.05 min^−1^mM^−1^ and 6.94 ± 0.3 min^−1^mM^−1^ for ethanol and butanol, respectively, indicating a significant (~6-fold) increase in the catalytic efficiency of *Th* ADH_ae in favor of butanol over ethanol.

**Figure 8:**
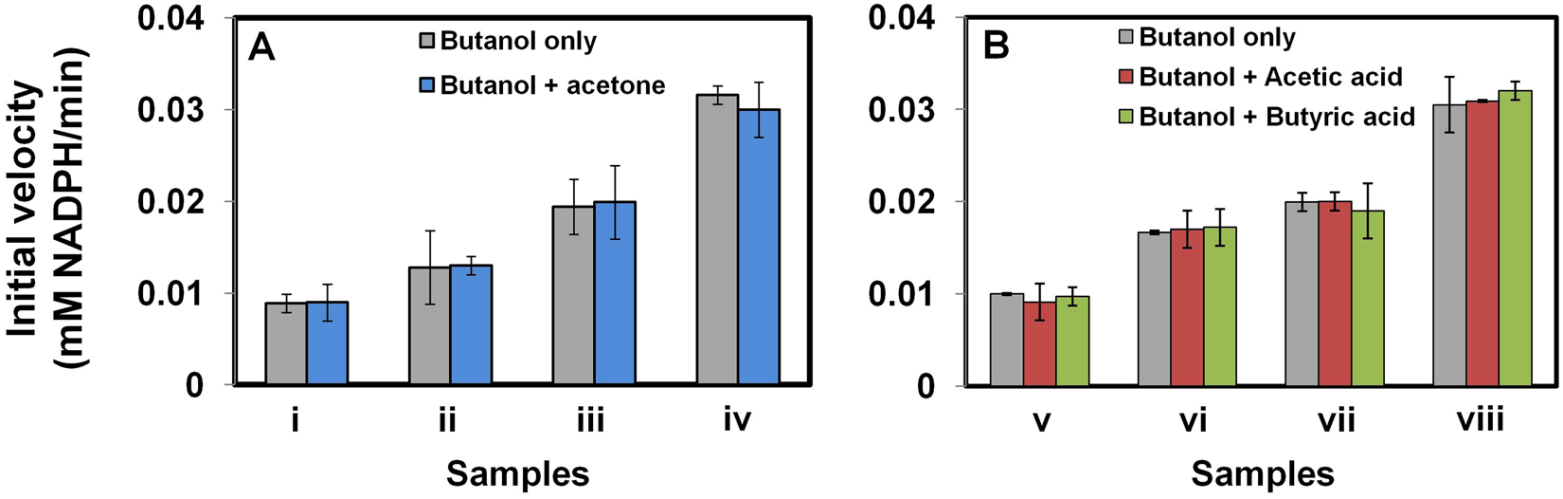
Effect of acetone, acetic acid and butyric acid on butanol activity of *Th* ADH_ae. (**A**) n-Butanol + acetone substrate mixtures contained: (A) 0.74 and 0.3 g/L, (B) 1.1 and 0.4 g/L, (C) 1.5 and 0.6 g/L, and (D) 3 and 1.2 g/L n-butanol and acetone, respectively. (**B**) n-Butanol + acetic or butyric acid samples contained: (A) 0.74 and 0.1 g/L, (B) 1.1 and 0.15 g/L, (C) 1.5 and 0.2 g/L, and (D) 3 and 0.4 g/L n-butanol and acid, respectively. Initial velocities of butanol-only sample and substrate mixtures were similar.

**Figure 9:**
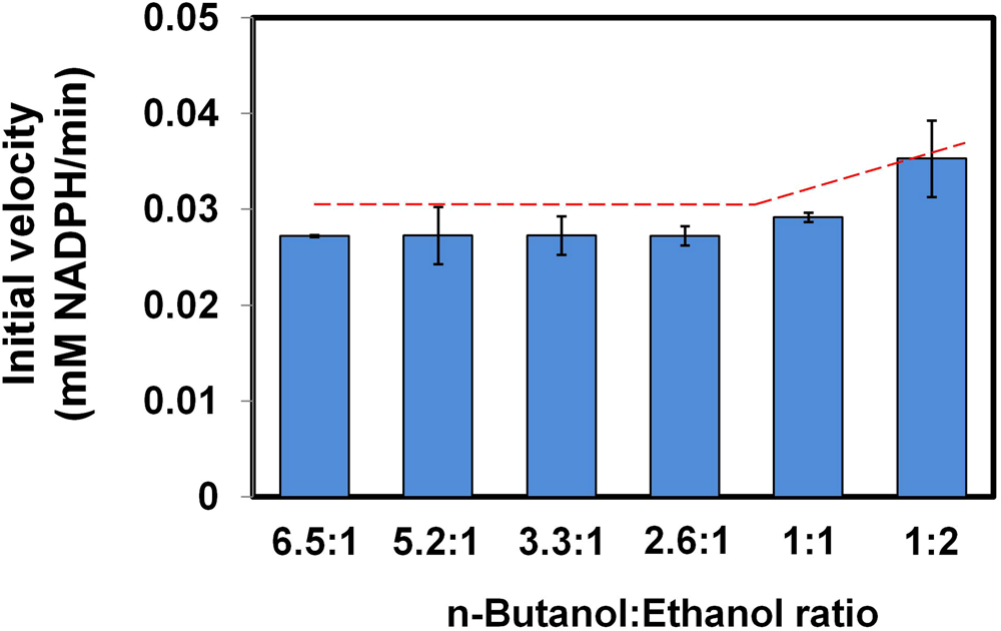
Effect of ethanol presence on the n-butanol activity of *Th* ADH_ae. Butanol was kept constant (2.6 g/L) while ethanol was varied, 0.4, 0.5, 0.8, 1, 2, and 5 g/L to yield butanol/ethanol ratios, 6.5:1, 5.2:1, 3.3:1, 2.6:1, 1:1, and 1:2, respectively. Initial velocities were similar at 6.5 to 2.6:1 n-butanol to ethanol ratio, indicating that *Th* ADH_ae activity on n-butanol was not affected by ethanol at these ratios. The dashed line indicates change in initial velocity as the concentration of ethanol increases.

Next, we investigated the effect of acetone, acetic acid, or butyric acid on the activity of *Th* ADH_ae with butanol as the primary substrate. Figure 8 shows that the presence of acetone, acetic acid, or butyric acid has no effect on *Th* ADH_ae activity towards butanol. The effect of ethanol on the activity of *Th* ADH_ae with butanol was also evaluated using butanol + ethanol mixtures containing 35 mM butanol and varying concentrations of ethanol (Fig. 9). The initial velocity of *Th* ADH_ae was constant at butanol:ethanol ratios of 6.5:1, 2.6:1 and even 1:1 but increased at 1:2 (Fig. 9); thus, ethanol interference does not register until we breach a 70 mM ethanol threshold, a finding consistent with the Km ~200 mM for ethanol. This result gains significance considering the amounts typical in ABE fermentation media (6 g/L or 81 mM butanol and 1 g/L ethanol or 22 mM). Unless genetic modifications lead to clostridial strains where ethanol production is enhanced by greater than 3.2-fold (i.e., 3.2 g/L) with no concomitant change in butanol production, our assay will accurately report on the butanol levels in the media.

**Table 2:** Comparison of n-butanol concentrations obtained from *Th* ADH_ae-based time-course butanol assay and gas chromatography-based analyses. The range of measurement is between 0.5 to 10 g/L.

***C. beijerinckii*** **culture supernatant**	**Butanol (g/L) as determined by gas chromatography**	**Butanol (g/L) as determined by** ***Th*** **ADH_ae assay**
A	8.1 ± 1.2	8.4 ± 2.5
B	6.4 ± 0.1	6.1 ± 2.4
C	7.2 ± 0.0	7.6 ± 1.9

As proof of principle, three random ABE fermentation supernatants generated from *Cb* fermentation of glucose media^11^ were screened for butanol using our *Th* ADH_ae-based assay. Butanol concentrations were determined from a calibration curve of known butanol concentrations versus absorbance, and compared with data obtained from gas chromatography analysis that is a proven benchmark. Table 2 shows that butanol concentrations obtained from the *Th* ADH assays (8.4, 6.1, and 7.6 g/L) parallel those obtained from gas chromatography (8.1, 6.4, and 7.2 g/L, respectively).

**Table 3:** Comparison of kinetic parameters of *Th* ADH_ae and *Th* ADH_an using two different end-point assay formats. k_cat_ and K_m_ values shown in the table represent the mean and standard error calculated from two independent experiments. K_m_ and V_max_ curve-fit errors of *Th* ADH_ae with butanol did not exceed 20% and 5.8%, respectively, for anaerobic mixing, and 17% and 11% for aerobic mixing. Similarly, K_m_ and V_max_ curve-fit errors of *Th* ADH_an with butanol did not exceed 25% and 7.7%, respectively, for anaerobic mixing, and 32% and 12%, for aerobic mixing. R^2^ values of individual trials exceeded 0.95.

**Kinetic parameters**	*Th* ADH_an		*Th* ADH_ae	
	**Aerobic mixing of reaction components**	**Anaerobic mixing of reaction components**	**Aerobic mixing of reaction components**	**Anaerobic mixing of reaction components**
*K*_m_ (mM)	22.3 ± 1.2^a^	12.8 ± 0.2^b^	30.2 ± 0.4^a^	12 ± 0.2^b^
*V*_max_ (mM NADPH/min)	0.006 ± 0.0003^a^	0.007 ± 0.0001^b^	0.013 ± 0.0001^a^	0.019 ± 0^b^
*k*_cat_ (min^−1^)	26.1 ± 1.1^a^	32 ± 0.2^b^	60.5 ± 0.5^a^	86.8 ± 0^b^
*k*_cat_/*K*_m_ (min^−1^ mM^−1^)	1.2 ± 0.1^a^	2.5 ± 0.1^b^	2 ± 0.01^a^	7.2 ± 0.2^b^

^a,b^Fisher’s LSD was applied to pair-wise comparison to separate means of kinetic parameters between aerobic and anaerobic assay conditions. Parameters with different letters in each row are significantly different at *p* < 0.05.

### *Th* ADH_an-based end-point assays can be adapted for high-throughput screening of *Cb* cultures for butanol

The *Th* ADH kinetic parameters described above were determined using initial velocity calculations. To simplify the assay for high-throughput, we employed an end-point measurement. Reaction mixtures assembled in microtiter PCR plates were incubated for 80 s at 80 °C using a thermocycler prior to absorbance measurements at room temperature using a regular 96-well plate. Reproducible *Th* ADH activity values were obtained with and without reaction quenching (data not shown), a result not entirely unanticipated given that the enzyme (derived from a thermophile) functions optimally at 80 °C and is inactive at room temperature. Therefore, we eliminated the quenching step. Using this simplified approach, we assessed the performance of Th ADH as described below.

We sought to compare *Th* ADH_ae and *Th* ADH_an activity using the end-point measurement with two formats: mixing the reaction components either under aerobic conditions or in an anaerobic chamber prior to sealing the PCR plate and performing the incubation for 80 s at 80 °C. This comparative analysis proved instructive. First, we compared the k_cat_ and K_m_ for *Th* ADH_ae that was determined using the time-course (Fig. 3) versus end-point (Table 3) measurements under anaerobic conditions. While the k_cat_/K_m_ was nearly the same, this concurrence obscured three-fold decreases in both k_cat_ and K_m_ using the end-point measurements. While time-course assays are more reliable than end-point measurements, it is also important to consider variations in heat-transfer (use of 1.5-ml tubes in a heat block for time-course versus a 96-well plate in a PCR cycler for end-point measurement). Second, regardless of whether we used *Th* ADH_ae and *Th* ADH_an, the k_cat_/K_m_ decreased by 2- to 3-fold when the reaction components were mixed under aerobic conditions prior to the incubation. Thus, while the ADH-based assay described here may be conducted outside the anaerobic chamber, the K_m_ is adversely affected by 2- to 3-fold (Table 3). Finally, although *Th* ADH_ae and *Th* ADH_an fared well when the assay components were mixed and assayed under anaerobic conditions, *Th* ADH_ae was slightly more active for unknown reasons (Table 3). Overall, it is clear that this end-point measurement-based anaerobic assay is well suited for a high-throughput platform to measure the concentration of butanol in multiple ABE fermentation samples. We have consistently observed that solventogenic *Clostridium* transformants even from the same transformation exhibit differences in cell growth, organic-acid re-assimilation and butanol production. While the basis is not well understood, non-uniformity in uptake of plasmids is a likely cause for such variability. Regardless of the underlying reason, this variability necessitates rapid screening methods for selecting transformants with desired phenotypes. Here, we sought to test the idea that our *Th* ADH_an-based butanol high throughput assay will help screen for hyper-butanologenic solventogenic *Clostridium* transformants. Here, we applied our assay to an ongoing metabolic engineering study where the objective is to increase NADPH regeneration and butanol production primarily by enhancing the ability of *Cb* to utilize glycerol as a co-substrate with glucose.

To accomplish our metabolic engineering goal, our work plan entailed the overexpression of two enzymes: glycerol dehydrogenase (encoded by *gldA*), which catalyzes the reduction of NADP^+^ to NADPH and the oxidation of glycerol to dihydroxyacetone (DHA); and dihydroxyacetone kinase (encoded by *dhaK*) that phosphorylates DHA to generate DHAP, which feeds into the glycolytic pathway. We constructed two plasmids containing either (1) two glycerol dehydrogenase (GDH)-encoding genes, *dhaD1* and *gldA1*, or (2) one GDH-encoding gene, *gldA1*, and one dihydroxyacetone kinase (DHAK)-encoding gene derived from the hyper glycerol-utilizing *Cp*. In plasmid 1, a Gly_5_ peptide linker was introduced between the two *Cp* GDH genes to tether the two proteins via their N- and C-terminal extended loops; the length of the linker (five residues) was chosen to minimize misfolding of the tandem proteins. Overexpression of *dhaD1* and *gldA1* as a fusion protein was expected to increase the local concentration of enzyme activity due to the proximity of the two active sites^18^. In plasmid 2, *gldA1* was linked to *dhaK* using a Gly_5_ peptide linker to enhance the conversion of reaction intermediates in the glycerol catabolism pathway and drive metabolic flux toward the glycolytic pathway.

Twenty-four single colonies (**A–X**) were randomly selected from two *C. beijerinckii* transformants, pWUR460_*dhaD1* + *gldA1* and pWUR460_*gldA1* + *dhaK*. The colonies were outgrown in 200-µL liquid culture in a microtiter plate and the supernatants screened for butanol using the *Th* ADH high throughput approach. Butanol concentrations were determined by extrapolation from the standard curve of known butanol concentration versus absorbance. As shown in Table 4, colonies B, C, G, J, K, and L from *Cb* pWUR 460_*dhaD1* + *gldA1* and Q, T, and X from *Cb* pWUR460_*gldA1* + *dhaK* produced up to 12 g/L of butanol.

**Table 4:** Use of *Th* ADH_an-dependent high-throughput assay to determine n-butanol concentrations in the culture supernatants of various recombinant Cb colonies (A - X).

**Colony ID [*dhaD1*** + ***gldA1*]**	**Butanol (g/L)**	**Colony ID [*gldA1*** + ***dhaK*]**	**Butanol (g/L)**
A	8.7 ± 0.02	P	2.0 ± 0.4
B	11 ± 0.5	Q	11.8 ± 0.12
C	11.8 ± 0.2	R	0.7 ± 0.02
D	6.9 ± 0.7	S	7.8 ± 1.5
E	1.6 ± 0.2	T	11.5 ± 0.2
F	4.3 ± 0.01	U	9.2 ± 0.12
G	12.7 ± 1.7	V	11 ± 0.3
H	4.3 ± 0.6	W	10.1 ± 2.9
I	3.8 ± 0.1	X	12.3 ± 0.02
J	11.4 ± 1.2		
K	12.1 ± 1.8		
L	11.6 ± 1		
M	2.9 ± 1		
N	3.4 ± 1.1		
O	4.3 ± 0.9		

## Conclusions

We developed a robust high-throughput spectrophotometric assay to directly measure butanol concentrations of microbial cell cultures. This assay utilizes a recombinant *Th* ADH that can accurately measure the amount of butanol present in cell cultures because the activity of the enzyme is not affected by the presence of other major ABE fermentation products. Notably, ethanol does not interfere with butanol measurements in this assay when the butanol:ethanol is > 2.6, a threshold fulfilled during native ABE fermentation. Therefore, our assay will complement efforts to metabolically engineer solventogenic *Clostridium* species for improved butanol production by facilitating rapid screening of mutant libraries of strains of interest. Protein engineering initiatives to further increase the catalytic specificity of *Th* ADH for butanol over ethanol and use of continuous assays (in an anaerobic setting) represent profitable future directions. Discovery or design of a *Th* ADH-like variant that can function under reduced temperature conditions also merits consideration.

## Electronic supplementary material

Supplementary materials
