# Supplementary material for: Development of a high-throughput assay for rapid screening of butanologenic strains
Source: Sci Rep. 2018 Feb 21;8:3379. doi: 10.1038/s41598-017-18074-7 (PMC5821843; doi:10.1038/s41598-017-18074-7)
Supplement: Supplementary file 1 — Supplementary materials [file 41598_2017_18074_MOESM1_ESM.pdf]

Supplementary Materials

Title: Development of a high-throughput assay for rapid screening of butanologenic strains

Authors: Chidozie Victor Agu, Stella M. Lai, Victor Ujor, Pradip K. Biswas, Andy Jones, Venkat Gopalan and Thaddeus Chukwuemeka Ezeji

Table S1: Overview of *Th* ADH n-butanol assay protocols highlighting improvements in protocol using microtiter plates.

Step	Time-course assay protocol for initial velocity determination	End-point assay protocol for endpoint measurements using microtiter PCR plates
1.	Prepare reaction mix on ice as follows: 15 µL unknown sample, 50 µL CAPS (500 mM, pH 11.6), 12.5 µL each of 10 mM DTT and 1 mM $FeCl_2$, 30 µL 2 mM $NADP^+$, and 5 µL *Th* ADH (10 µM).	Prepare reagent master mixes. Master mix 1 = 25 µL of 500 mM CAPS (pH 11.6), 6.25 µL each of 10 mM DTT and 1 mM $FeCl_2$. Master mix 2 = 15 µL 2 mM $NADP^+$ and 1.5 µL 10 µM *Th* ADH.
2.	Label five 1.5-mL eppendorf tubes: 0, 20, 40, 60, or 80 s.	Aliquot 7.5 µL of unknown samples and butanol standards (10 to 25 mM) + 1 µL distilled water into microtiter PCR plate.
3.	Aliquot 25-µL reaction mix into each tube.	Add 37.5 µL of master mix 1 and 16.5 µL master mix 2.
4.	Incubate each tube on a heat block for the time indicated.	Incubate at 80˚C for 80 s. A thermocycler is used for this step.
5.	After each incubation, quench reaction with 25 µL 1 M NaOAc + 1 mM EDTA, pH 4.5.	Transfer reactions into wells of a flat-base microtiter plate.
6.	Read Abs_{340} immediately.	Read absorbance at 340 nm.
7.	Repeat steps 3 to 6 for each time point.	Interpolate unknown concentration from standard curve.
8.	Prepare reaction mixes for each butanol standard as in (1) above.	
9.	Repeat steps 2 to 6 for each standard reaction mix.	
10.	Transfer each 50 µL reaction left on ice as in (6) above into wells of a flat-base microtiter plate.	
11.	Read absorbance at 340 nm.	
12.	Interpolate unknown concentration from standard curve.	

Table S2: List of primers and PCR protocols used to generate inserts.

S/N	Insert	Primers (5' to 3') and PCR strategy
1.	[*dhaD1+gldA1*]	dhaD1-F: ATAGGGCCC**AGGAGG**TATCCATGGATGAGAAAAGCATTTATTTGTCC<br>dhaD1-R: ACCACCACCACCACC TTTACACATCCTCTTCTTTTCC<br>gldA1-F: AAA GGTGGTGGTGGTGGTATGAGTTATAGTGTTTTTTTACCAAG<br>gldA1-R1: TAAAAAAATAAGAGTTACCATTTATTAGACAGCTTTCACAGGC<br>gldA1-R2:<br>CGACCTCGAGAATTCACTATGAAACAATATTAAAAAAATAAGAGTTACCATTTATTAGA<br><br>**PCR strategy:** (1) *dhaD1* was amplified from *C. pasteurianum* genomic DNA using nested PCR with primer pair dhaD1-F & R (AT1 = 54˚C, AT2 = 71˚C) (2) *gldA1* was also amplified from *C. pasteurianum* genomic DNA with primer pair gldA1-F & gldA1-R1 (AT1 = 54˚C, AT2 = 66˚C), then re-amplified the resulting amplicon with gldA1-F & R2 (AT1 = 52˚C, AT2 = 67˚C). (3) *dhaD1* and *gldA1* amplicons were fused via two step SOE-PCR, Step 1: *dhaD1* and *gldA1* served as both primers and template (forward and reverse templating fragments, respectively) AT of overlap region = 59˚C. In Step 2 SOE-PCR, the amplicon from step 1 was used as template for normal PCR using primers dhaD1-F and gldA1-R2 (AT = 67˚C).
2.	[*gldA1+dhaK*]	gldA1-F: ATAGGGCCCAGGAGGTATCCATGGATGAGTTATAGTGTTTTTTTACCAAG<br>gldA1-R: ACCACCACCACCACC GACAGCTTTCACAGGCTCATTAC<br>dhaK-F5: GTCGGTGGTGGTGGTGGTATGAAAAAGATAATAAATAAACCAG<br>dhaK-R1: TAAAAAAATAAGAGTTACCATTTACTACTTTATAACCTCTGAAATC<br>dhaK-R2:<br>CGACCTCGAGAATTCACTATGAAACAATATTAAAAAAATAAGAGTTACCATTTACTACT<br><br>**PCR strategy:** (1) *gldA1* was amplified from *C. pasteurianum* genomic DNA using nested PCR with primer set gldA1-F and gldA1-R (AT1 = 54˚C, AT2 = 71˚C), (2) *dhaK* was also amplified from *C. pasteurianum* genomic DNA using primer set dhaK-F5 and dhaK-R1 (AT1 = 50˚C, AT2 = 62˚C), then re-amplified using primers dhaK-F5 and dhaK-R2 (AT1 = 50˚C, AT2 = 67˚C). (3) Amplicons from (1) and (2) above were spliced by two-step SOE PCR. Step 1: AT = 63˚C; Step 2: AT = 67˚C.
